# Expansions and contractions in gene families of independently-evolved blood-feeding insects

**DOI:** 10.1186/s12862-020-01650-3

**Published:** 2020-07-17

**Authors:** Lucas Freitas, Mariana F. Nery

**Affiliations:** 1 Laboratório de Genômica Evolutiva, Departamento de Genética, Evolução, Microbiologia e Imunologia, Instituto de Biologia, Universidade Estadual de Campinas, Rua Bertrand Russell, S/N, Cidade Universitária, IB, Bloco H, Campinas, SP Brazil; 2 School of Biological and Chemical Sciences, Queen Mary University of London, London, UK

**Keywords:** Comparative genomics, Hematophagy, Adaptation, Evolution, Heat-shock protein, Chemosensory protein

## Abstract

**Background:**

The blood-feeding behavior evolved multiple times in Insecta lineages and it represents an excellent opportunity to study patterns of convergent molecular evolution regarding this habit. In insects the expansion of some gene families is linked with blood-feeding behavior, but a wide study comparing the evolution of these gene families among different lineages is still missing. Here we gathered genomic data from six independently-evolved hematophagous lineages, aiming to identify convergent expansions and/or contractions of gene families in hematophagous lineages of insects.

**Results:**

We found four rapidly evolving gene families shared by at least two hematophagous independently-evolved lineages, including a heat-shock and a chemosensory protein. On the expression of these four rapidly evolving gene families we found more genes expressed in mated individuals compared with virgin individuals in rapidly-expanded families and more genes expressed in non-blood-feeding individuals compared with blood-feeding individuals in rapidly-contracted families.

**Conclusion:**

Our results reveal a new set of candidate genes to be explored in further analysis to help the development of new strategies to deal with blood-feeding vectors and also presents a new perspective to study the evolution of hematophagy identifying convergent molecular patterns.

## Background

Hematophagy, or the habit of blood-feeding, is a widespread feeding behavior, being found in several groups like insects, fishes, mammals, and birds [1]. Several of these hematophagous species are able to transmit viruses, bacteria, and parasites during the blood-feeding behavior, which are harmful to the host, causing huge economic loss and health impacts on humans and animals. For instance, only on the American continent Dengue fever has an estimated cost of $2.1 billion per year, in 2010 US dollars [2]. Further, more than 17% of all infectious diseases are carried by vectors, causing 700,000 deaths annually [3].

The main group responsible for vector-borne diseases is the class Insecta, where hematophagy independently evolved multiple times [4, 5]. To better understand the basis of insects’ hematophagy, different approaches have been employed in many fields of science, e.g. animal behavior [6], ecology [7], biochemistry [8] and genomics [9, 10].

Comparative functional genomics studies in hematophagous insects usually target salivary glands tissues due to their specific adaptations to hematophagy, as reviewed by Arcà & Ribeiro (2018) [11]. For example, proteins found in salivary glands of hematophagous insects have anti-clotting and anti-itching properties allowing them to pierce other animals and feed on blood without being detected [12].

Since hematophagy evolved independently among insects, it is expected that distinct proteins would be found in salivary glands of independently-evolved lineages, which was reviewed by Ribeiro et al. (2010) [12]. However, some of these distinct proteins have the same function: lipocalins and D7 proteins act as a biogenic amines scavenger in ticks, kissing bugs and mosquitoes [13], revealing a functional convergence related with hematophagy.

Still, other kinds of convergent evolution are found at molecular level. Convergent amino acid substitutions [14], convergent shift in evolutionary rates [15] and convergence in gene families copy number variation [16] are examples of convergent molecular evolution that are reported in the literature. In this context, the independent evolution of hematophagy in insects represents an excellent opportunity to study patterns of convergent molecular evolution. Although several strategies to infer convergent molecular evolution are being developed and used [17], few works aimed to study the convergence of gain and loss in gene copies number across distinct lineages [16]. For example, in insects, the expansion of some gene families has been linked to blood-feeding behavior, since the expression of extra copies of genes would help in blood digestion [18–21].

Despite the importance of gene family turnovers, a wide study comparing the evolution of gene families among different blood-feeding lineages of insects is still missing. Accordingly, here we gathered genomic data from six independently-evolved hematophagous lineages, Culicidae, Psychodidae, Glossinidae (Diptera order), Reduviidae, Cimicidae (Hemiptera order) and Phthiraptera (Psocodea order), aiming to identify convergent expansions and/or contractions in gene families in hematophagous lineages of insects and their role in the evolution of the blood-feeding behavior using gene expression analysis.

## Results

**Fig. 1:**
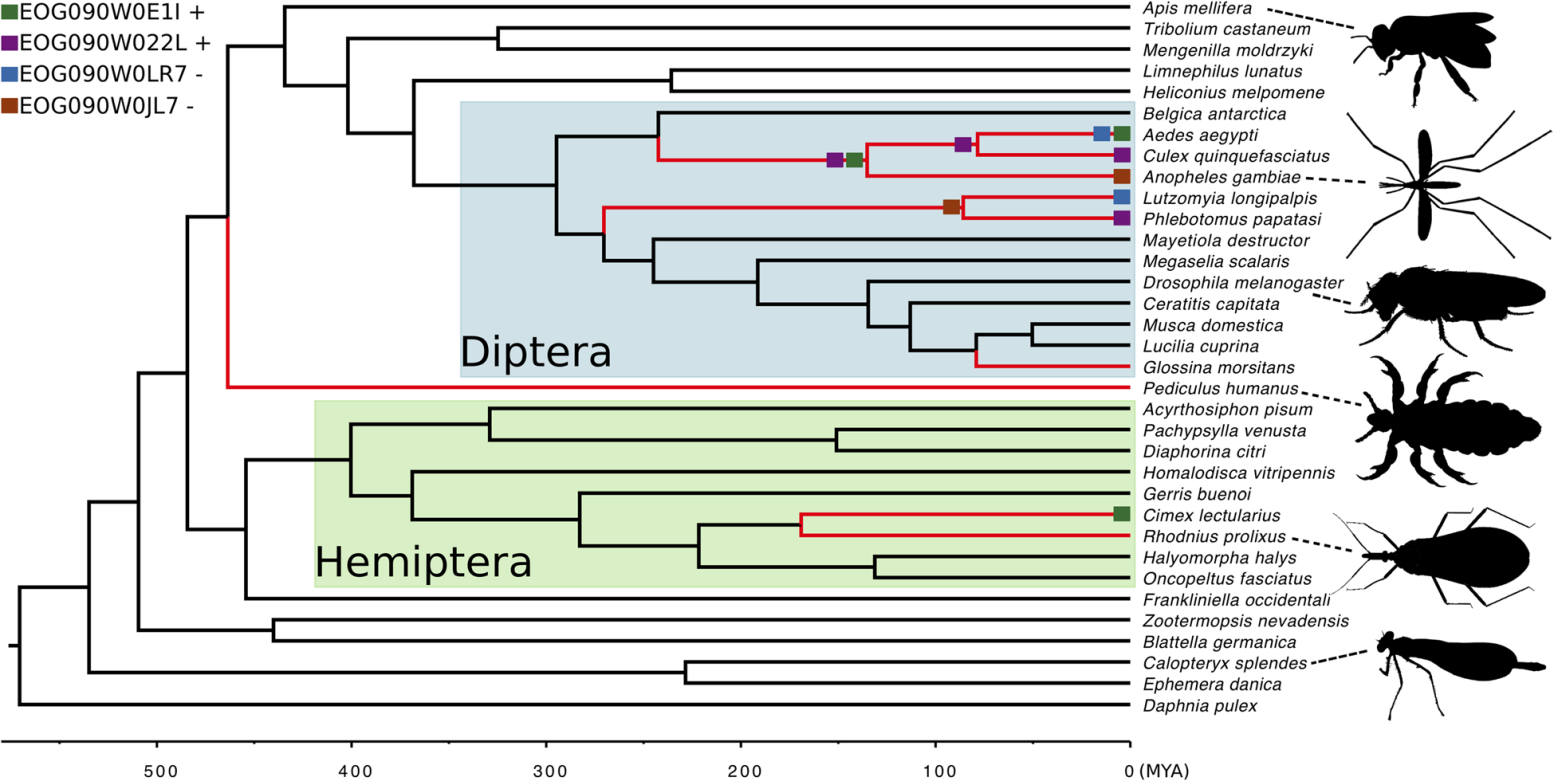
Insecta phylogenetic timetree used in this work. Red branches represent six independently-evolved hematophagous lineages. *Rhodnius prolixus* and *Cimex lectularius* represent two independent events of hematophagous evolution, the non-hematophagous genomes of their sister groups were not available. Diptera (blue box) and Hemiptera (green box) are the groups with multiple hematophagous lineages. Green and purple squares indicate lineages with rapid expansions in multiple hematophagous lineages, while blue and brown squares indicate lineages with rapid contractions in multiple hematophagous lineages

Regarding the inference of gene families evolution, CAFE estimated an error distribution (e) of 0.075 which means that less than 10% of gene families in our dataset have the wrong number of gene copies. Using this error distribution, we tested two models using the timetree inferred with MCMCtree: the global model, where the whole tree has the same λ rate, and the hemato model, where hematophagous branches have a distinct λ rate compared with non-hematophagous branches (Fig. 1). The λ rate for the global model was 1.7 × 10^− 3^, while this same λ rate was 1.2 × 10^− 3^ for hematophagous branches and 1.7 × 10^− 3^ for non-hematophagous branches in the hemato model. The likelihood-ratio test was significant (*p* = 0), showing that the hemato model fits better the data compared with the global model. This result shows that hematophagous lineages have a slower rate of gain and losses of genes compared with non-hematophagous lineages. Although hematophagous lineages had an overall slower rate of gain and losses, they have approximately the same mean number of gene families with rapid expansions (14.92 ± 14.56) compared to non-hematophagous lineages (14.6 ± 21.15), per lineage. On the other hand, hematophagous lineages had a significantly higher number of rapid contractions (8.83 ± 8.25) compared to non-hematophagous lineages (4.92 ± 6.75), per lineage (Wilcoxon rank sum test *p*-value = 0.015).

Since we aimed to identify gains/losses in independently-evolved hematophagous lineages, we focused on rapidly evolving families that were found in at least two of these lineages. We found four of such gene families: rapid expansions in a small heat shock protein HSP20 (EOG090W0E1I) and in a carboxylesterase (EOG090W022L), and rapid contractions in a transmembrane protein (EOG090W0LR7) and in an odorant-binding protein (EOG090W0JL7) (Table 1), but only the HSP20 showed a convergent pattern of expansion considering a non-hematophagous ancestor. As control of this test, we applied the same procedure in nearby non-hematophagous lineages; in this case, only two genes, a glucose-methanol-choline oxidoreductase (EOG090W08UU) and an uncharacterized protein (EOG090W00HB), had rapid expansions in distinct orders and no multiple contractions were found. Another interest gene family was the EOG090W0B2X family, a carbohydrate kinase, which was present in 44 branches but missing in all hematophagous lineages.

**Table 1:** Number of gene gain (+) and loss (−) (in parenthesis) and functional annotation for all rapidly-evolving gene families. In bold, rapid/expansions in independently-evolved hematophagous lineages

OrthoDB ID	Lineage (gene change)	Functional annotation
EOG090W0E1I	***Aedes aegypti*(+ 8), Culicidae (+ 2),*Cimex lecturianus*(+ 2)**, *Culex quinquefasciatus* (− 7)	Metal ion binding
EOG090W022L	**Culicinae (+ 4),*Culex quinquefasciatus*(+ 3),*Phlebotomus papatasi*(+ 4), Culicidae (+ 4)**, *Lutzomyia longipalpis* (− 12)	Carboxylic ester hydrolase activity
EOG090W0LR7	***Aedes aegypti*(− 3),*Lutzomyia longipalpis*(− 4)**, *Homoladisca vitripennis* (+ 3), *Lucilia cuprina* (+ 3), *Culex quinquefasciatus* (+ 2), *Zootermopsis nevadensis* (+ 2)	–
EOG090W0JL7	***Anopheles gambiae*(− 12), Psychodidae (− 8)**, *Musca domestica* (+ 6), *Aedes aegypti* (+ 11), *Culicinae* (+ 7), *Culicidae* (+ 4), *Heliconius melpomene* (+ 3)	Chemosensory protein

**Fig. 2:**
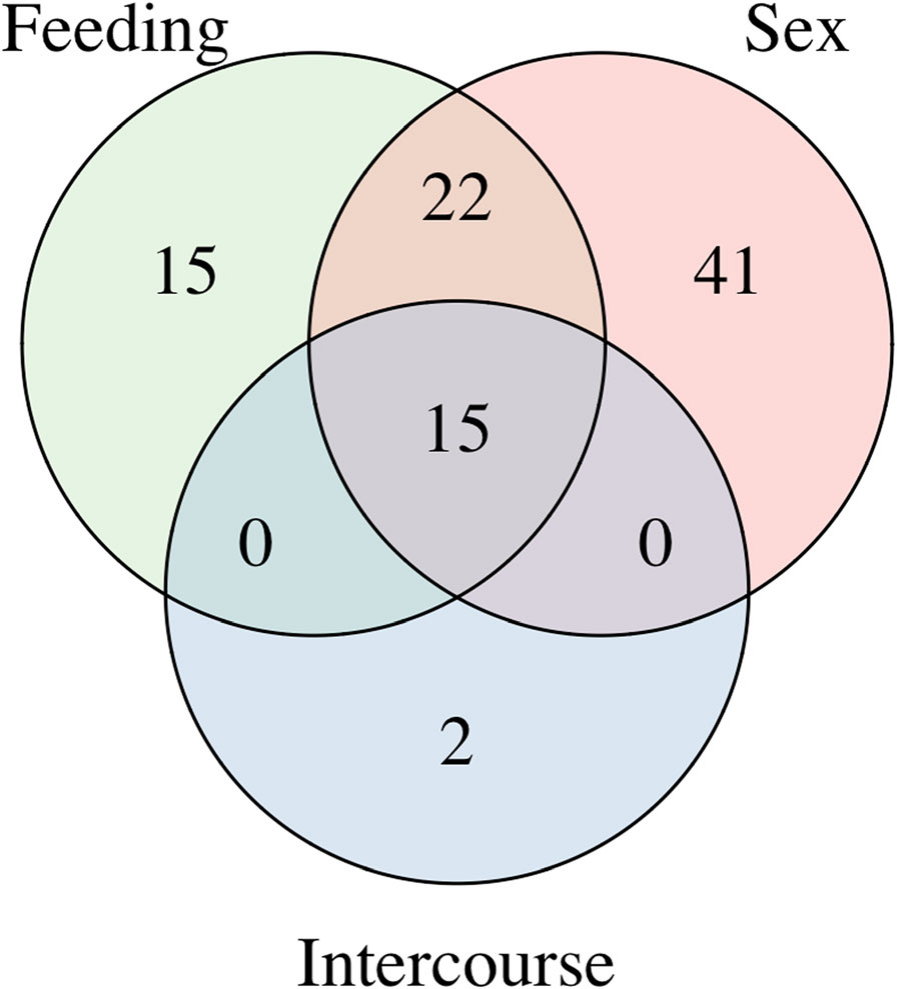
Venn diagram showing the number of genes found in the feeding (blood-feed vs. non-blood-feed individuals), sex (female vs. male) and intercourse (mated vs. virgin individuals) gene sets and the genes shared between them

To deep explore the role of these four candidate gene families in the hematophagous behavior in Insecta, we specifically searched for genes differentially expressed in the transcriptomes available at VectorBase. From 315 genes in all hematophagous species in these four gene families, we were able to identify 95 genes differentially expressed in VectorBase database (Additional file 1: Tables S1-S4). From these 95 genes, 78 were found in the sex set, 17 in the intercourse set, 52 were found in the feeding set, 22 genes were shared between sex and feeding and 15 genes were shared by all categories (Fig. 2).

**Fig. 3:**
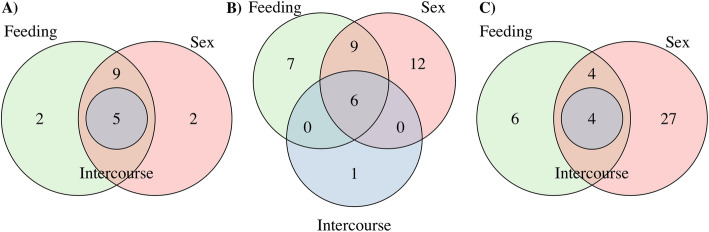
Number of genes differentially expressed in all sets tested for gene families A) HSP20, B) Carboxylesterase and C) Odorant-binding protein. The transmembrane protein is not present because it was found being differentially expressed only in the intercourse set

On gene families with rapid expansions, the HSP20 family contains 18 genes differentially expressed (Additional file 1: Table S1), among which five are shared by all sets, nine are shared between sex and feeding, two are exclusively found in the feeding set and other two are exclusively found in the sex set (Fig. 3A). The carboxylesterase family contains 33 genes differentially expressed (Additional file 1: Table S2), including six shared by all sets, nine shared by sex and feeding sets, 12 exclusively found in the sex set, seven in the feeding set and one found in the intercourse set (Fig. 3B). Functions of these gene families were assigned as metal ion binding and carboxylic ester hydrolase activity for HSP20 and carboxylesterase families, respectively.

Among gene families with rapid contractions, the transmembrane protein EOG090W0LR7 contains only one gene differentially expressed, AGAP009139, found in the intercourse set, and present both in mated and virgin individuals. The odorant-binding EOG090W0JL7 contains 41 genes differentially expressed (Additional file 1: Table S4), but only four are shared between all sets, while the other four genes are shared between sex and feeding sets. In the sex set, we found 27 exclusive genes, while the feeding set contains six exclusive genes (Fig. 3C). Regarding the function of these families, both the transmembrane protein EOG090W0LR7 and the odorant-binding EOG090W0JL7 do not have functional annotation in VectorBase, thus we blasted them against InterPro [22] and Pfam [23] databases to assign their functions. Both families remained without a functional annotation, however, the odorant-binding EOG090W0JL7 was assigned as a member of the chemosensory protein family (CSP) based on OrthoDB annotation.

## Discussion

Shifts in the numbers of copies from a specific gene family represent a raw source of evolutionary innovation. More copies of a specific gene could increase the content of protein, allowing the organism to better deal with some tasks, like digestion [24] or detoxification of some compound [25]. On the other hand, some gene losses leading to phenotypic and metabolic changes are also thought to be adaptive [26]. In common, both expansions and contractions might indicate an adaptation to a new lifestyle.

The transition to hematophagy represents a new lifestyle, where insects independently developed new physiological adaptations to seek blood sources and to feed on blood [27, 28]. Here we explored the evolution of gene families in Insecta to identify significant convergent patterns of expansions and contractions in gene families of six independently-evolved blood-feeding lineages and their relationship with hematophagy.

We found four rapidly evolving families in at least two hematophagous independently-evolved lineages. Gene family EOG090W0E1I rapidly expanded independently in Culicidae and Cimicidae orders and it was the only true example of convergent evolution, where the expansion occurred after the transition to hematophagy. According to UniProt annotation, it belongs to the HSP20 family. Previous works showed high expression levels of other HSP, HSP70, after blood-feeding in mosquitoes, kissing bugs and bed bugs, suggesting that HSP protects their midguts from the stress caused by the warm blood ingested [29, 30].

Gene family EOG090W022L also rapidly expanded independently in Culicidae and Psychodidae orders and, according to UniProt annotation, it is a carboxylesterase. Carboxylesterase is a detoxification gene previously associated with resistance to insecticide in mosquitoes [31, 32] and is hypothesized that this expansion may help these groups to detoxify the huge amount of amino acids and heme following a blood-feeding [33].

Notably, our results show that hematophagous species have a slower λ rate than non-hematophagous species, contrary to the assumption that a transition to a new lifestyle would favor expansions and/or contractions. However, it is likely that many non-hematophagous species present a great number of expansions and contractions due several reasons. For example, the milkweed bug genome [34] shows expansions in the number of gene copies related to CSPs and retention of enzymes involved in the amino acid metabolism.

On the expression patterns of rapidly-evolved genes found in VectorBase and their role in the evolution of hematophagy, our results are unexpected. VectorBase data rely on Culicidae expression studies, thus we would expect more genes overexpressed in females, mated and blood-feeding insects [35]. However, from all sets, only intercourse set showed more expressed genes in the expected category, mated individuals. The other two sets, feeding, and sex, showed more expressed genes in the unexpected categories of male and non-blood-feeding individuals, respectively (Table 2).

**Table 2:** Number of overexpressed genes found in VectorBase according to categories and sets

	Sex			Intercourse			Feeding		
	Female	Both sets	Male	Mated	Both sets	Virgin	Blood	Both sets	Non-blood
Rapid evolving families	13	4	26	9	2	1	9	11	18
Slow evolving families	10	16	9	2	2	1	2	1	11

Although expansions in salivary gland proteins in hematophagous insects are well reported in literature [9, 10, 36], none of the two expanded genes families found in this work are a classic salivary gland protein. A possible explanation is that we focused our searches on rapid expansions and contractions shared by two or more independently-evolved blood-feeding lineages, and salivary gland genes expansions are usually specific to each lineage [11, 12]. Besides it, the orthology assessment from OrthoDB may differ from the orthology assessment of salivary gland proteins, which is a well-studied group of proteins regarding hematophagous species [11] and where orthology is usually manually reviewed [37]. Indeed, from the 53 salivary proteins distributed in 24 gene families found in 19 *Anopheles* genomes by Àrca et al. (2017) [37], OrthoDB distributes them in 38 orthologous groups (gene families), which probably decreased turnovers rates inferred by CAFE for salivary proteins in our study. Only two salivary proteins from Àrca et al. (2017) [37] are not present in OrthoDB: a cE5-Anophelin and a hyp8.2.

Regarding the two gene families with rapid contractions, their functions are not well-annotated as gene families with rapid expansions. The only annotation for EOG090W0LR7 classified it as an integral component of membrane, while EOG090W0JL7 is annotated as a CSP but none function is associated with it. Assuming its role as a CSP, we cannot state that the contraction inferred in EOG090W0JL7 represents an adaptation. For example, chemosensory genes have a wide variation in the number of copies in Insecta, ranging from 1 to 265, where expansions and contractions both happened several times [38]. Even the number of CSPs copies found within Culicidae have a large variation: 6.8 ± 1.1 CSPs were found in 19 species of the Anophelinae sub-family, while 51 ± 28.8 CSPs were found in three species in the Culicinae sub-family [39]. On the expression patterns of slowly-evolved genes found in VectorBase and their role in the evolution of hematophagy, Table 2 does not show a clear pattern. Only the feeding set displays differences between distinct categories, showing more overexpressed genes in non-blood-feeding individuals compared with blood-feeding individuals.

Previously, several works explored the relationship between gene families expansions and hematophagy [10, 20, 37, 40], but they usually focused only in one species/group or few gene families and they did not employ a statistical approach to model the evolution of gene families. Two exceptions are the works of Neafsey et al. (2015) [9] and Thomas et al. (2020) [41]. The first described the evolution of gene families within Anophelinae and found a λ rate of 3.2 × 10^− 3^ along the genome, estimating a relatively conserved number of CSP across this group.

Compared with Neafsey et al. (2015) [9], our λ rate for hematophagous lineages is approximately three times lower and we did find a contraction in CSP for *Anopheles gambiae*. However, our dataset spans a much deeper time-scale and the contraction in *A. gambiae* represents the loss from the ancestor of Anophelinae and Culicinae, which is not the case of Neafsey et al. (2015) [9]. Thomas et al. (2020) [41], who also explored a deep-time scale of insects, inferred the λ rate for several families (Table S13 on their work), where the mean found was 1.18 × 10^− 3^, similar to our estimates (1.12 × 10^− 3^ and 1.17 × 10^− 3^ for hematophagous and non-hematophagous lineages, respectively).

## Conclusions

In this work, we investigated the evolution of gene families as a first approach to identify convergent evolution in blood-feeding insects. The significant turnover in gene numbers found in four gene families in more than one hematophagous lineage indicates evidence of convergent molecular evolution related to blood-feeding. Following this line, we also would expect these genes to be differentially expressed after blood-meal, intercourse or in females. Here we found more genes expressed in mated individuals compared with virgin individuals in rapidly-expanded families and more genes expressed in non-blood-feeding individuals compared with blood-feeding individuals in rapidly-contracted families.

Beyond revealing a new set of candidate genes to be explored in further analysis to help the development of new strategies to deal with blood-feeding vectors, our findings suggest that the same strategy could be efficiently used in more than one vector, due to convergent evolution, and also present a new perspective to study the evolution of hematophagy identifying convergent molecular patterns.

## Methods

### Genomic data set

All data used in this study was obtained through OrthoDB v. 9.1 [42]. We generated single-copy-only gene families (one gene copy per species per gene family) and a multiple-copies gene family matrix (one or more gene copies per species per gene family) for Metazoa. The first was used to estimate the divergence time, while the latter was used to estimate changes (gain and loss) in the evolution of gene families. We selected 33 Arthropoda species (Fig. 1) plus *Daphnia pulex* as an outgroup. We did not include *Bactrocera* and *Polypedilum* genus because other species within their families were already present.

### Divergence time estimation

We used Misof et al. (2014) [4] phylogenetic tree as input to our order-relationships. Within Diptera and Hemiptera orders, the relationships were based on Song et al. (2016) and Weirauch et al. (2019) [43, 44]. From the 38,842 orthologous groups (OGs) available at OrthoDB we filtered out species with multiple copies and aligned each OG with MAFFT v7.407 (FFT-NS-1 algorithm) [45]. Then we removed gap-rich regions with Gblocks v. 0.91b (minimum length of block = 5 and up to 50% gap positions were allowed) [46]. We only selected genes where more than 50% of amino acid sites were kept by GBlocks and more than 17 species were present in the alignment, resulting in a concatenated matrix of 683 genes and 288,500 amino acid sites.

Using this assembled tree and alignment we used MCMCtree v. 4.9 h [47] to perform a Bayesian divergence time estimation with the approximate likelihood calculation described in dos Reis & Yang (2011) [48]. Analyses were performed twice to check MCMC convergence, and fossil calibration priors followed Misof et al. (2014) [4].

### Gene families evolution

We used CAFE v. 4.1 [49] to identify gene families with rapid expansions and/or contractions in copy numbers along hematophagous-only lineages for the 38,842 OGs available at OrthoDB. Using the timetree estimated earlier and the number of gene copies per family, CAFE first estimates the distribution of error (e) in the number of gene copies present in the dataset. Then, accounting to e, CAFE infers the birth and death (λ) rate, which represents the average rate of genomic turnover (gains and losses) per gene per million years [50].

In this work, we compared a global model, with one λ rate for all branches, and the other with two λ rates, split between hematophagous and non-hematophagous branches. To compare both models and infer which one fits better the data, CAFE employs a set of simulations for the global model. For each simulation, it performs a likelihood-ratio test (LRT) that will be used to draw a null distribution where the real LRT will be compared. Finally, we annotated the function of these genes using UniProt [51] and VectorBase [52] databases. We also used VectorBase to identify which of these genes were statistical differentially expressed in transcriptomes according to three sets: feeding (blood-feed vs. non-blood-feed individuals), intercourse (mated vs. virgin individuals) and sex (female vs. male) patterns, all of them related with the blood-feeding behavior [35]. *p*-values < 0.05 according each study present in VectorBase were considered significant.

## Supplementary information

**Additional file 1.**

## Data Availability

The datasets generated and/or analysed during the current study are available in https://github.com/freitas-lucas/GenFamBlood.

## Abbreviations

HSP20: Small heat shock protein HSP20
CSP: Chemosensory protein family
OG: Orthologous group
